# Short-term effects of mobilization on oxygenation in patients after open surgery for pancreatic cancer: a randomized controlled trial

**DOI:** 10.1186/s12893-021-01187-2

**Published:** 2021-04-07

**Authors:** Monika Fagevik Olsén, Suada Becovic, Elizabeth Dean

**Affiliations:** 1 Department of Health and Rehabilitation/Physiotherapy, Institute of Neuroscience and Physiology, Sahlgrenska Academy, University of Gothenburg, Gothenburg, Sweden; 2 Department of Surgery, Institute of Clinical Sciences, Sahlgrenska Academy, University of Gothenburg, Gothenburg, Sweden; 3 Department of Physical Therapy, Faculty of Medicine, University of British Columbia, Vancouver, Canada; 4 Department of Physical Therapy, Sahlgrenska University Hospital, SE 431 45 Gothenburg, Sweden

**Keywords:** Mobilization, Pancreatic surgery, Post-operative

## Abstract

**Background:**

Despite the unequivocal role of progressive mobilization in post-surgical patient management, its specific effects and timing, particularly after abdominal surgery, remain debated. This study’s aim was to examine the short-term effects of mobilization on oxygenation in hemodynamically stable patients after open surgery for pancreatic cancer.

**Methods:**

A randomized controlled clinical trial was conducted in which patients (n = 83) after open pancreatic surgery were randomized to either the same-day mobilization group (mobilized when hemodynamically stable within four hours after surgery) or the next-day mobilization group (mobilized first time in the morning of the first post-operative day). Mobilization was prescribed and modified based on hemodynamic and subjective responses with the goal of achieving maximal benefit with minimal risk. Blood gas samples were taken three times the evening after surgery; and before and after mobilization on the first post-operative day. Spirometry was conducted pre-operatively and on the first post-operative day. Adverse events and length of stay in postoperative intensive care were also recorded.

**Results:**

With three dropouts, 80 patients participated (40 per group). All patients in the same-day mobilization group, minimally sat over the edge of the bed on the day of surgery and all patients (both groups) minimally sat over the edge of the bed the day after surgery. Compared with patients in the next-day mobilization group, patients in the same-day mobilization group required lower FiO_2_ and had higher SaO_2_/FiO_2_ at 1800 h on the day of surgery (p < .05). On the day after surgery, FiO_2,_ SaO_2_/FiO_2,_ PaO_2_/FiO_2_, and alveolar-arterial oxygen gradient, before and after mobilization, were superior in the same-day mobilization group (p < 0.05). No differences were observed between groups in PCO_2_, pH, spirometry or length stay in postoperative intensive care.

**Conclusions:**

Compared with patients after open pancreatic surgery in the next-day mobilization group, those in the same-day mobilization group, once hemodynamically stable, improved oxygenation to a greater extent after mobilization. Our findings support prescribed progressive mobilization in patients after pancreatic surgery (when hemodynamically stable and titrated to their individual responses and safety considerations), on the same day of surgery to augment oxygenation, potentially helping to reduce complications and hasten functional recovery.

*Trial registration:* This prospective RCT was carried out at the Sahlgrenska University Hospital, Sweden. The study was approved by the Regional Ethical Review Board in Gothenburg (Registration number: 437-17). Trial registration: “FoU in Sweden” (Research and Development in Sweden, URL: https://www.researchweb.org/is/vgr) id: 238701 Registered 13 December 2017 and Clinical Trials (URL:clinicaltrials.gov) NCT03466593. Registered 15 March 2018.

## Background

Commensurate with the evolution of space science including microgravity-simulation bed-rest studies and exercise science, the multisystem effects of recumbency and inactivity were well documented several decades ago [1–14]. Despite this unequivocal knowledge base, its translation to the mobilizing of acutely ill patients, particularly those requiring high dependency care such as intensive and postoperative care, has lagged [15]. Over the past 20 years, interest has resurged with a call for further elucidation of the role of mobilization in acutely ill patients based on randomized controlled clinical trials (RCTs).

Mobilization is the term that has emerged in the literature that refers to patients being ‘upright and moving’ during hospitalization [16, 17]. It subsumes a prescriptive and progressive series of steps in which patients move from recumbency to eventual walking with varying levels of assistance depending on the patient’s status and responses. Additional exercises can be prescribed at each step as part of the functional return continuum toward the patient’s achieving maximal and speedy recovery.

The hemodynamic and pulmonary consequences of positioning patients upright have long been known to be profound [2, 18]. Superimposing the acute effects of exercise augments these benefits [19]. Collectively, this body of physiologic knowledge supports that being ‘upright and moving’ is the de facto ‘physiologic’ body position for humans. With respect to the hemodynamic and pulmonary consequences of bed rest, supine alters chest wall and hemi-diaphragmatic configuration, in turn, intrathoracic and intraabdominal pressures, in turn affecting cardiopulmonary function, fluid shifts and hemodynamics [20, 21]. Functional residual capacity (FRC) and lung compliance are reduced, and airway resistance increased [18], predisposing the patient to airway closure, increased work of breathing, and decreased arterial oxygen pressure. These effects are accentuated with anesthesia and abdominal surgery [22] contributing to greater risk of complications, especially in patients undergoing open abdominal and thoracic procedures or high-risk patients, e.g., overweight individuals, smokers, and those with pulmonary diseases [23]. In addition, physical deconditioning associated with immobility during bed rest, begins rapidly [14].

The effects of changing body position on arterial oxygen saturation (SaO2) and blood gases after surgery have been investigated [24, 25]. In one review [25], 12 articles were identified in which the short-term effects of body position changes were evaluated after abdominal surgery. No RCTs were there identified in which the short-term effects of progressive mobilization from sitting to standing on blood gases were evaluated compared to an un-mobilized reference group. Sitting and semi recumbent positions improved postoperative pulmonary function compared with supine in half of the studies.

Despite recognition clinically of the need for patients to be upright and moving, the level of evidence of the short-term effects of mobilization on oxygenation and its isolated effect on postoperative cardiovascular and pulmonary complications warrants elucidation. Research and debate have largely focused on when to mobilize patients, specifically, ‘early’ vs. ‘late’, and issues related to safety and cost benefit [26–28]. The challenges appear to be in defining ‘early’ vs. ‘late’ and even what constitutes mobilization. Agreed and shared definitions are essential for the advancement of practice for acutely-ill patients and related research [29].

So-called ‘early’ intensive mobilization is a component of enhanced recovery after surgery (ERAS). ERAS is a multimodal and multi-professional approach to the management of surgical patients [30, 31]. Because of its approach, the effect of mobilization per se is confounded [30–32]. Nonetheless, the evidence supporting ‘early’ mobilization is graded as ‘strong’ [31, 32].

Protocols for ‘early’ mobilization not included within ERAS after abdominal and thoracic surgeries have also been evaluated. In one review [33], 8 such articles were retrieved, of which 6 were RCTs. None of these trials evaluated the short-term effects of mobilization on oxygenation, a fundamental outcome of care after anesthesia and intensive care. Rather, their outcomes included length of hospital stay, physical activity, patient-reported outcomes, and safety. The review concluded little evidence exists to guide clinicians in effective ‘early’ mobilization to improve function and related outcomes [33].

We concur that RCTs have a role in examining the short-term effects of mobilization on oxygenation with respect to its relative timing, i.e., earlier rather than later, in patients undergoing various types of surgery, particularly high-risk surgery. However, such trials need to incorporate reflexivity with respect to the clinician’s clinical decision-making processes in progressing patients. Even with the experimental control required in RCTs, individual differences among patients exert profound effects (e.g., age, health status, lifestyle practices, pre-morbid conditioning, arousal, and medications). Such reflexivity in the clinician’s clinical decision-making is aimed at achieving the maximal benefit with least risk, as well as deciding when to rest the patient or discontinue mobilization.

To reconcile apparent disparities between long-standing physiologic and experimental literature within a rigorous RCT framework, this study’s aim was to compare short-term oxygenation effects of mobilization initiated the same day of surgery with those on the day after surgery, in patients after open surgery for pancreatic cancer. We examined the effect of initiation of mobilization as soon as the patient had stabilized after surgery, and then systematically progressed patients through the steps of mobilization according to their responses and safety considerations. In this way, we anticipated minimizing recumbency and bed rest for each patient and augmenting hemodynamic and ventilatory adaptation to being progressively upright and moving, requirements for eventual maximal functional return.

## Methods

### Study setting and design

To calculate sample size for the trial, the power analysis was based on a difference in arterial partial pressure of oxygen (PaO_2_) of 1 kPa, a standard deviation of 1.5 kPa, power of 0.80, and alpha of 0.05. The sample size was estimated to be 36 participants per group. To adjust for participant drop-outs, sample size was increased by 10%, thus 40 participants were recruited to each group.

A consecutive series of 123 adult patients scheduled for open, radical pancreatic surgery between December 2017 and May 2019 were invited to participate at their pre-operative visit to the out-patient clinic scheduled the day before surgery. Exclusion criteria included cognitive impairment or inability to communicate in Swedish. The patients were given verbal and written information and gave their written consent to participate.

**Fig. 1:**
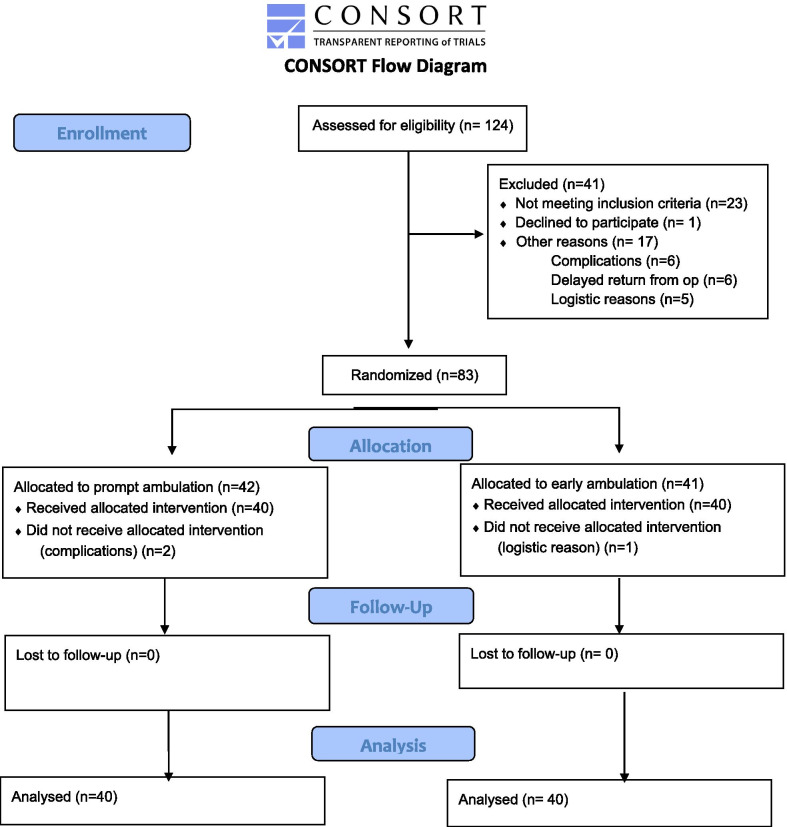
Consort flow diagram

Of the 123 patients who were assessed for eligibility, 41 were excluded (Fig. 1). There were no differences in baseline characteristics between those who completed the trial and those who were excluded. The remaining 83 patients were randomly allocated to either the experimental group or the reference group, specifically, 42 were allocated to the same-day mobilization group and 41 to the next-day mobilization group. Randomization was performed when the patients returned from the operating room using opaque sealed envelopes prepared by a person independent from the study and based on a computerized random number table. The allocation to each group was 1:1. Three patients did not receive the allocated intervention, two because of complication (one with pain because of a nonfunctional epidural anesthesia and one because of a postoperative bleeding) and one because of logistic reasons, thus were excluded from the analysis. Characteristics of the remaining 80 patients (40 per group) appear in Table 1. There were no differences in demographic data between groups except concerning length of anesthesia (difference 48 min, p = 0.045).

**Table 1:** Demographic data for the patients

Demographic variable	Measure or unit	Same-day mobilization group n = 40	Next-day mobilization group n = 40	p-value between groups
Age	Years	66.8 (8.0)	68.0 (10.2)	0.560
Sex	Male	26 (65)	19 (48)	0.176
Body mass index	kg/m^2^	25.3 (4.2)	25.3 (4.1)	0.977
Lung disease	Yes	2	0	0.494
Smoking history	Yes	24 (60)	30 (75)	0.334
	No	6 (15)	3 (8)	
	Ex-smoker*	10 (25)	7 (18)	
Heart disease	Yes	0	0	n.a
American Society Anesthesiology risk classification, 1–5: 1 = slight, 2 = minimal, 3 = moderate	1	11 (28)	9 (22)	0.160
	2	24 (60)	19 (48)	
	3	5 (12)	12 (30)	
WHO, preoperative performance score	Asymptomatic, n	23	22	0.664
	Symptomatic, ambulant, n	14	9	
	Symptomatic, < 50% in bed, n	0	3	
	Symptomatic, > 50% in bed, n	0	2	
	Missing, n	3	4	
Preoperative chemotherapy	Treatment, n	2	4	0.432
Tumor classification	T1/T2/T3/T4, n	2/7/12/12	0/10/14/5	0.337
	N0/N1/N2, n	13/13/6	10/12/11	0.389
	M0, n	33	32	n.a
	Missing or benign, n	7	8	–
Surgery type	Whipple, n	36	35	0.737
	Total pancreatectomy, n	4	5	
Length of surgery	Hours	6.5 (1.3)	7.2 (1.8)	0.065
Length of anesthesia	Hours	8.0 (1.5)	8.8 (1.9)	0.045
Vascular resection	None, n	33	27	0.322
	Venous wedge, n	2	7	
	Venous end-to-end, n	4	5	
	Arterial + venous	1	1	
Perioperative vasoactive drugs	Number of patients, n	40	40	1.000

Mean (SD) or number (%). *n.a. *non applicable

*Cessation of smoking > 8 weeks prior the surgery

### Surgical procedures

The planned surgical interventions were either Whipple’s procedure or total pancreatectomy performed through a median incision. During a Whipple procedure, the head of the pancreas, the distal part of the ventricle, the first part of the small intestine (duodenum), the gallbladder, and the bile duct are removed. Anastomoses are created to connect the remaining parts of the pancreas, ventricle, and intestine. Patients who underwent Whipple surgery had two site drains and those who underwent total pancreatectomy had one drain. All patients had nasogastric tube inserted and catheter à demeure.

Short-acting anesthetics and analgesics were used consistent with practice standards. Induction of anesthesia was performed with propofol, remifentanil, and rocuronium, and maintained with supplemental oxygen, air, and sevoflurane. Norepinephrine was administered via a central line to maintain mean arterial pressure above 70 mm Hg during surgery and throughout the immediate post-operative period. All patients had a thoracic epidural inserted before induction of anesthesia. In the epidural, a mix of bupivacaine, fentanyl, and epinephrine was administered throughout the surgical procedure. Post-operatively, patient-controlled analgesia was administered through the thoracic epidural cannula.

Patients who did not undergo the planned procedure, required prolonged mechanical ventilation, or arrived in the post-operative ward late at night were excluded from further participation in the trial. Other exclusion criteria were complications with bleedings or analgesia control immediately after surgery.

### Intervention

Before the mobilization intervention in either group, each patient’s bedside nurse optimized analgesia, and other drug administration to minimize hemodynamic instability and discomfort based on standard practices. This was defined as systolic blood pressure less than 90 mm Hg, ≤ 8 or ≥ 30 breaths/minute, ≤ 90 or ≥ 130 heart beats/minute, or insufficiently rousable. Blood pressure, heart rate, and SaO_2_ were monitored by IntelliVue MP2 (Phillips Healthcare, Amsterdam, The Netherlands) via an arterial cannula (CODAN Medizinische Geräte GmbH & Co KG, Lensahn, Germany).

For continuity of the study protocol often throughout a prolonged day, patients were mobilized in the postoperative intensive care unit by experienced intensive care health professionals, i.e., physiotherapists and registered nurses. Before the study was initiated, they were familiarized with the study protocol and criteria for mobilizing for maximal therapeutic benefit and safety. This ensured that mobilization was tailored according to each patient’s physical and hemodynamic status and progressed according to their responses in terms of therapeutic response and safety.

Patients in the same-day mobilization group, were mobilized, when hemodynamically stable, for the first time within the first four hours after surgery, i.e., from lying, to side lying, to sitting over the edge of the bed, to standing, to walking beside the bed, to transferring and sitting in a chair, to walking in the cubicle area [34]. When resting in bed, patients were recumbent with head of bed elevated to 60°.

Patients in the next-day mobilization group began mobilization for the first time on the morning after surgery, by which time they were hemodynamically stable. When lying in bed the head of bed was elevated to 30° according to usual clinical practice.

On the morning after surgery, all patients were progressively mobilized. This was performed according to the same principles as applied on the day of surgery. The patients in both groups were mobilized according to the schedule included in our ERAS program, during that and forthcoming days of hospitalization. This includes tailored progressed mobilization according to the patient´s physical and hemodynamic status at least one hour (in total) the first postoperative day, 2 h the second postoperative day, 3 h the third postoperative day and four hours the fourth postoperative day. The mobilization was mixed by semi-recumbent cycle ergometry once the third postoperative day and at least twice daily thereafter.

In addition, patients in both groups performed the same breathing exercises with positive expiratory pressure (PEP) which was prescribed and daily supervised by a physiotherapist. The PEP/RMT system (Mediplast, Malmö, Sweden) was used with mid-expiratory pressure of + 10 cm of water. The training was performed in 3 sessions of 30 breaths at least 8 times/day.

### Outcome measures

Blood gas samples were taken from the arterial cannula (BD FloSwitch, Becton Dickson BD Medical, Singapore) at 1800, 2000 and 2200 h on the day of surgery, and before and after mobilization on the first day after surgery. Samples were analyzed immediately by RAPID Point® 500 (Siemens Healthineers, Munich, Germany) and SaO_2_, arterial oxygen partial pressure (PaO_2_), arterial carbon dioxide partial pressure (PaCO_2_), and pH level were recorded. Samples were taken while the patient was breathing ambient room air. If supplemental oxygen was required, its level was recorded. Consequently, PaO_2_ and SaO_2_ are reported as PaO_2_/FiO_2_ (primary outcome) and SaO_2_/FiO_2._ To assess oxygen levels, the degree of alveolar shunt and $\dot{V}$/$\dot{Q}$ mismatch, and the alveolar-arterial O_2_ (A-a O_2_) gradient were calculated.

Lung function, i.e., forced vital capacity (FVC), forced expiratory volume in one second (FEV_1_) and peak expiratory flow (PEF), measured with spirometry EasyOne (ndd Medical Technologies Inc., MA, US), was assessed during the pre-operative visit and on the first post-operative day. Pre-operatively, lung function assessment was performed in a standardized sitting position according to international guidelines [35]. Post-operatively, lung function was similarly measured before the mobilization intervention. Body position was however standardized to semi-sitting in bed with head of bed elevated to 60°.

Duration of each step in the progressive mobilization sequence (e.g., sitting over the edge of the bed or in a chair and time standing/walking) was recorded. Reasons for terminating mobilization or any adverse events were also recorded. Masking the intervention to either the patients or health professionals was not feasible.

Additional data recorded from the patient charts included demographics, time (hours) in the post-operative intensive care unit, days in hospital after surgery, and complications including postoperative pneumonia.

All methods were carried out in accordance with relevant guidelines and regulations.

### Statistical analysis

Group assignment was blinded for outcome analyses and participant data were coded. Differences between groups were analyzed using independent t-tests and chi square tests. Differences within the group as a whole were tested with paired t-tests. For comparison between groups over time for non-normally distributed variables, the Fisher´s non-parametric permutation test was used for continuous variables. The confidence interval for the mean difference between groups was also based on Fisher’s non-parametric permutation test. For comparisons between groups over time for normally distributed variables, a mixed model with a compound symmetry covariance pattern was used. Alpha was set at 0.05.

## Results

### Mobilization same day of surgery

Based on their assessed physiological readiness to be mobilized and responses to it, patients in the same-day mobilization group all minimally sat over the edge of the bed within four hours after surgery, Table 2. Mobilization was initiated within the first three hours after surgery. Average time sitting was six minutes (SD 4.3, range 2 to 12). All but two of the patients were mobilized to standing and stood an average of 90 s. One patient transferred to a chair and sat for 20 min. Indications for discontinuing the progressive and response-driven mobilization session were blood pressure drop (n = 11), vertigo (n = 4), nausea/vomiting (n = 6), pain (n = 6), fatigue (n = 3), anxiety (n = 1), muscle weakness (n = 2), and disorientation (n = 1).

**Table 2:** Type and duration of mobilization after pancreatic surgery. Mean (± SD)

Mobilization		Same-day mobilization group (n = 40)	Next-day mobilization group (n = 40)	p-value
Day of surgery	Time sitting over the edge of the bed, min	6.0 (4.3) n = 40	–	–
	Time sitting in a chair, min	20 n = 1	–	–
	Time standing, sec	43.4 (53.2) n = 38	–	–
Day after surgery	Time sitting over the edge of the bed, min	5.5 (2.6) n = 40	6.1 (2.5) n = 40	0.298
	Time sitting in a chair, min	77.3 (48.6) n = 33	56.8 (52.1) n = 29	0.071
	Time standing, sec	81.3 (63.8) n = 40	83.8 (69.8) n = 39	0.868

### Mobilization the day after surgery

On the day after surgery, all patients were mobilized given they were hemodynamically ready and based on their responses, Table 2. Patients in the same-day mobilization group sat over the edge of the bed for an average of 5.5 min compared to patients in the next-day mobilization group who sat for 6.1 min (p = 0.298). Additionally, 33 of the 40 patients in the same-day mobilization group sat in a chair for an average of 77 min compared to 29 of the 40 patients in the next-day mobilization group (p = 0.295) who sat for an average of 57 min (p = 0.071). Reasons for discontinuing the progressive response-driven mobilization included blood pressure drop (n = 12), vertigo (n = 3), nausea/vomiting (n = 3), pain (n = 6), fever (n = 1), muscle weakness (n = 1), and effects of sepsis (n = 1).

### Blood gas analyses

Results of the blood gas analysis appear in Tables 3 and 4. The same-day mobilization group required lower levels of supplementary oxygen at the first assessment time (1800 h on the day of surgery) and before being mobilized on the first post-operative day (p = 0.012 and 0.009, respectively). The SaO_2_/FiO_2_ was higher in the same-day mobilization group at 1800 h the day of surgery, and before and after mobilization on the first post-operative day (p = 0.008, 0.003 and 0.002, respectively). The PaO_2_/FiO_2_ was higher after the mobilization session on the first post-operative in the same-day mobilization group (p = 0.037). The A-a O_2_ gradient was lower for the same-day mobilization group both before and after being mobilized on the first post-operative day (p = 0.016 and 0.003, respectively). No differences were observed between groups for either PCO_2_ or pH (p > 0.05) (Table 3).

**Table 3:** FiO_2_, SaO_2_/FiO_2_, PaO_2_/FiO_2_ and A-a O_2_ gradient of patients in the two groups

Variable	Follow-up	Same-day mobilization group (n = 40)	Next-day mobilization group (n = 40)	p-value	Difference between groups Mean (95% CI)
FiO_2_, %	POD 0: 1800 h	23.1 (3.6) n = 32	26.0 (4.3) n = 25	0.012	0.029 (0.008; 0.050)
	POD 0: 2000 h	25.0 (5.8) n = 39	27.1 (6.0) n = 36	0.13	0.021 (− 0.005; 0.049)
	POD 0: 2200 h	23.3 (4.7) n = 38	24.8 (6.2) n = 39	0.24	0.015 (− 0.010; 0.040)
	POD 1 before and after mobilization	21.0 (0.0) n = 34	22.6 (3.5) n = 39	0.009	0.016 (0.005; 0.028)
SaO_2_/FiO_2_	POD 0: 1800 h	421.7 (51.9) n = 32	379.8 (62.1) n = 25	0.008	− 41.9 (− 71.6; − 11.2)
	POD 0: 2000 h	401.1 (73.3) n = 39	369.5 (70.9) n = 36	0.060	− 31.6 (− 65.0; 1.4)
	POD 0: 2200 h	423.2 (59.4) n = 38	402.2 (69.0) n = 39	0.16	− 21.0 (− 50.5; 8.8)
	Before mobilization POD 1	450.2 (8.5) n = 34	424.3 (52.7) n = 39	0.003	− 25.8 (− 44.4; − 8.0)
	After mobilization POD 1	451.7 (7.9) n = 34	426.0 (50.5) n = 39	0.002	− 25.7 (− 43.3; − 8.5)
PaO_2_/FiO_2_	POD 0: 1800 h	353.6 (57.2) n = 32	342.1 (74.1) n = 25	0.51	− 11.5 (− 46.3; 23.2)
	POD 0: 2000 h	357.6 (51.6) n = 39	367.4 (120.8) n = 36	0.63	9.82 (− 32.65; 51.49)
	POD 0: 2200 h	365.3 (77.2) n = 38	348.0 (65.0) n = 39	0.31	− 17.3 (− 49.0; 14.2)
	POD 1: Before mobilization	346.4 (48.7) n = 34	322.9 (64.5) n = 39	0.085	− 23.6 (− 50.9; 3.4)
	POD 1: After mobilization	362.3 (41.6) n = 34	339.1 (50.5) n = 39	0.037	− 23.1 (− 44.7; − 1.4)
A-a O_2_ gradient	POD 0: 1800 h	5.06 (3.35) n = 32	6.60 (4.10) n = 25	0.12	1.54 (− 0.45; 3.50)
	POD 0: 2000 h	5.92 (3.83) n = 39	7.18 (6.49) n = 36	0.31	1.25 (− 1.16; 3.70)
	POD 0: 2200 h	4.95 (3.92) n = 38	6.55 (5.24) n = 39	0.13	1.60 (− 0.46; 3.70)
	POD 1: Before mobilization	4.34 (1.51) n = 34	6.02 (3.78) n = 39	0.016	1.68 (0.31; 3.10)
	POD 1: After mobilization	3.88 (1.30) n = 34	5.45 (2.99) n = 39	0.003	1.58 (0.49; 2.69)

Mean (SD) / n is presented as mean difference (95% confidence interval)

*POD* post-operative day, *FiO*_*2*_ % fraction of inspired supplemental oxygen, *PaO*_*2*_*/FiO*_*2*_ partial pressure of arterial blood over fraction of inspired supplemental oxygen, *A-a O*_*2*_* gradient* alveolar arterial oxygen gradient

**Table 4:** PaCO_2_ and pH of patients in the two groups

Variable	Follow-up	Same-day mobilization group (n = 40)	Next-day mobilization group (n = 40)	p-value	Estimated effect of same-day mobilization (95% CI)
PaCO_2_, kPa	POD 0: 1800 h	4.8 (0.4) n = 32	5.1 (0.5) n = 25	0.28	− 0.128 (− 0.361; 0.104)
	POD 0: 2000 h	4.8 (0.5) n = 39	4.8 (0.5) n = 36	0.57	0.060 (− 0.150; 0.270)
	POD 0: 2200 h	4.9 (0.4) n = 38	4.9 (0.5) n = 39	0.65	− 0.048 (− 0.256; 0.160)
	POD 1: Before mobilization	4.7 (0.3) n = 34	4.7 (0.4) n = 39	0.64	0.050 (− 0.162; 0.261)
	POD 1: After mobilization	4.8 (0.5) n = 34	4.8 (0.5) n = 39	0.76	− 0.033 (− 0.246; 0.179)
	Overall estimated effect of same-day mobilization			0.80	− 0.020 (− 0.177; 0.137)
pH	POD 0: 1800 h	7.39 (0.3) n = 32	7.38 (0.04) n = 25	0.18	0.013 (− 0.005; 0.031)
	POD 0: 2000 h	7.39 (0.04) n = 39	7.38 (0.04) n = 36	0.16	0.006 (− 0.011; 0.023)
	POD 0: 2200 h	7.40 (0.03) n = 38	7.39 (0.04) n = 39	0.47	0.012 (− 0.005; 0.028)
	POD 1: Before mobilization	7.43 (0.04) n = 34	7.43 (0.04) n = 39	0.16	0.004 (− 0.013; 0.020)
	POD 1: After mobilization	7.41 (0.03) n = 34	7.40 (0.04) n = 39	0.67	0.013 (− 0.004; 0.029)
	Overall estimated effect of same-day mobilization			0.18	0.009 (− 0.004; 0.023)

Mean (SD) and mean difference (95% confidence interval); *POD* post-operative day

To further elucidate patients’ oxygenation responses to mobilization on the first post-operative day, the results of the two groups were pooled. There were differences in PaO_2_/FiO_2_ of − 16 [95% CI 5.0;27.1, p = 0.005]; A-a O_2_ gradient of − 0.51 [− 0.86;− 0.17, p = 0.004] and pH of − 0.02 [− 0.03;− 0.01, p < 0.001]; but no changes in SaO_2_/FiO_2_ of 1.6 [− 0.01;0.15, p = 0.095] or of PaCO_2_ 0.05 [− 0.05;0.15, p = 0.327].

### Spirometry, length of stay, and adverse events

Lung function, i.e., FVC, FEV_1_ and PEF (% of predicted values) measured with routine spirometry decreased for both groups after surgery (p < 0.001), however there were no differences between groups (Table 5). In addition, there were no differences between groups with respect to number of patients who developed pneumonia, the length of stay in the post-operative intensive care unit or in hospital (Table 5). No adverse events such as falls or accidental drain removal during mobilization were recorded.

**Table 5:** Spirometric measures, number of pneumonia cases, time in post-operative care unit and length of stay after surgery

Spirometric variables	Measurement recording time	Same-day mobilization group n = 40	Next-day mobilization group n = 40	p-value
FVC, % predicted	Pre-operatively	98.0 (16.2)	94.5 (17.6)	0.361
	Post-operatively	59.2 (18.9)	58.3 (19.3)	0.824
FEV_1_, % predicted	Pre-operatively	91.2 (15.8)	90. 8 (20.2)	0.922
	Post-operatively	50.9 (13.9)	51.6 (18.2)	0.836
PEF, % predicted	Pre-operatively	89.2 (24.5)	83.5 (28.2)	0.333
	Post-operatively	43.2 (19.1)	41.2 (18.7)	0.642
Pneumonia cases, n		0	3	0.116
Time in post-operative intensive care, h		21.6 (5.2)	22.9 (10.9)	0.423
Length of stay after surgery, days		11.1 (5.2)	10.6 (4.1)	0.707

Mean (SD) or n

*FVC* forced vital capacity, *FEV*_*1*_ forced expiratory volume in one second, *PEF* peak expiratory flow

## Discussion

This RCT is the first of its kind in which oxygenation variables were compared between two groups of patients who had undergone open upper abdominal surgery, namely, those who were mobilized for the first time on the same-day of pancreatic surgery, and those who were mobilized for the first time the day-after surgery, in a response-driven manner.

The findings of our trial were unequivocal. Compared with patients in the next-day mobilization group, patients in the same-day mobilization group had higher SaO_2_/FiO_2_ levels and lower A-a O_2_ gradients the day after surgery. They required less supplemental oxygen than those in the next-day mobilization group. In addition, patients in the same-day mobilization group, had higher values for PaO_2_/FiO_2_ on the first post-operative day that those in the next-day mobilization group.

Mobilizing patients for the first time on the day after surgery has been studied previously. In a Japanese study, Kaneda and colleagues [36] examined whether walking within our hour of surgery was safe after lobectomy in patients with lung cancer. In that retrospective study, 36 patients who were mobilized out of bed on the same day as their surgery and 50 patients who were up and walked on the next day after surgery were included. The investigators concluded that same-day mobilization was safe in terms of no adverse events for that patient cohort with no falls or drainage tubes displacement, and no patient reports of untoward pain. In addition to being safe, the intervention was shown to have several therapeutic benefits. Patients who were mobilized on the same day as surgery had a shorter period of requiring supplemental oxygen and fewer of them had a PaO_2_/FiO_2_ ratio of less than 300 on the third post-operative day. These findings are consistent with ours.

In our study, patients in the same-day mobilization group minimally sat over the edge of the bed on the day of surgery based on our progressive mobilization protocol that was informed by patients’ readiness and responses (therapeutic and safe). The time sitting averaged 6 min. Only one patient in the group was mobilized to sitting in a chair at bedside. On the day after surgery, all patients in both groups were actively mobilized. Time sitting over the edge of the bed was 5 to 6 min and 62 patients also transferred to a chair. No adverse effects related to falls or accidental drain removals occurred during the mobilization sessions in either group. Progressive mobilization was guided by patient response, thus was modified, or discontinued due to untoward decreases in blood pressure; and reports of nausea, undue pain and fatigue that were considered clinically important.

In both groups in our study, spirometry was measured pre-operatively and again post-operatively before the mobilization session on the first post-operative day. There were no differences in the spirometric variables between groups either pre-operatively or post-operatively. Previous trials evaluating the effects of breathing exercises after abdominal surgery using spirometric measures as outcomes have reported comparable findings [37–40]. The lack of an apparent effect on lung volumes may reflect less sensitivity of spirometric variables to detect improvement in pulmonary function post-operatively compared with blood gases.

Arguments and barriers against ‘early’ mobilization have been raised vis-à-vis time and effort weighed against benefit [41, 42]. In the initial post-operative period, there is increased risk of hemodynamic instability and compromised oxygenation. Thus, patients need to be closely monitored to maximize the response-driven progression of mobilization and its benefits, and to minimize adverse effects. Compared with patients in the next-day mobilization group, patients in the same-day mobilization group tolerated being out of bed longer the day after surgery which is associated with other physiological benefits including increased arousal, hemodynamic and pulmonary function, earlier normalization of bowel and bladder function, and reduced effects of bed rest deconditioning [43, 44]

In the present study, PaO_2_ is reflected in both the PaO_2_/FiO_2_ and the A-a O_2_ gradient. The healthy range for PaO_2_/FiO_2_, the Horovitz Index or Quotient, is between 350 and 450. A value below 300 is considered the threshold for mild lung deficiency, 200 moderately severe, and below 100 severe deficiency [45]. The average PaO_2_/FiO_2_ across both groups in our study never dropped below the criterion for mild injury. The PaO_2_ was also reflected in the A-a O_2_ gradient. This gradient represents the extent of $\dot{V}/\dot{Q}$ mismatch. There were differences between the groups, all favoring the same-day mobilization group. This supports that mobilization had reduced $\dot{V}/\dot{Q}$ mismatch in that group.

Our findings help to reconcile the apparent disconnect between the physiologic evidence and experimental evidence with respect to getting surgical patients upright and moving. Amundadottir and colleagues [46] recently provided some explanation regarding this disconnect between the physiologic literature supporting the harmful effects of bedrest and immobilization, and the clinical experimental literature describing the importance of mobilization, yet its underuse. They queried the appropriateness of RCT designs for interventions such as mobilization [47]. They argued that clinically mobilization is initiated based on patients’ readiness with respect to their hemodynamic stability and progression of the intervention based on patient responses, therapeutic benefit, and safety considerations, rather than being initiated at a prescribed time, i.e., a pre-set time for early mobilization, or with a tightly structured protocol. RCTs, they argued, may not allow sufficient reflexivity for the clinician to progress the patient based on therapeutic responses or modify the intervention. Hence, in tightly controlled trials, some patients may be undertreated (insufficient gravitational and exercise stimuli) and others potentially pushed to less safe limits.

There are several strengths of our trial. As various surgical procedures affect patients in distinct ways, only patients undergoing open pancreatic surgery were recruited. This is a procedure with length of anesthesia lasting approximately 8 h and surgery duration 6 to 7 h; both factors contribute to greater post-operative risk.

To control for potential variability across hospital settings in clinical practices and procedures, our trial was undertaken in one large regional and university hospital, thus was a single center trial which limited potential cross center variability. Being a regional hospital, the hospital has a large referral base such that many surgeries are conducted each year which maximizes the experience and expertise of the multi-professional team members. In addition, the possibility of mobilizing a patient in the immediate post-operative period can be affected by several factors, e.g., circulatory, respiratory, and analgesic, thus requires a high degree of expertise from those physiotherapists and nurses who performed the mobilization intervention in our study, to walk the fine line between therapeutic benefit and safety.

There are some limitations of our study. First, to estimate the requisite sample size, power analysis was based on a difference in PaO_2_ of 1 kPa. This difference was somewhat arbitrary; however, it was chosen to be able to evaluate the acute effect of our intervention on PaO_2_ and its indices. The intention to take blood gas samples while patients were breathing room air was not possible in all patients. Therefore, the presentation of the SaO_2_ and PaO_2_ has been adjusted based on the level of supplemental oxygen. On the other hand, calculation of SaO_2_/FiO_2_, PaO_2_/FiO_2_ and A-a O_2_ gradient enabled us to expand our variables to reflect O_2_ uptake and $\dot{V}/\dot{Q}$ mismatch. Another limitation is that 6 (4.8%) patients who were eligible to participate were excluded because they arrived too late in the evening for the mobilization intervention to be initiated. In an optimal situation, mobilization should be performed irrespective of time, however this was not feasible in our study based on late-night staffing constraints. Of interest is the time between the patient’s arrival at the postoperative unit and when mobilization was initiated. Examining this variable in future trials will enable us to analyze the effect of this variable specifically. In addition, in our trial, patients who underwent two types of pancreatic procedure were included, given that both are associated with high risk of postoperative complications. However, there are differences between the procedures and their indications. Although this difference may have influenced our findings, the proportion of patients who underwent each type of surgery was comparable between groups.

Future studies are needed to replicate and extend our findings. First, long-term extension studies are needed to establish the degree to which these short-term oxygenation benefits in response to same-day mobilization translate into fewer complications, earlier discharge, and faster progression along the recovery trajectory and long-term functional recovery. Second, this trial needs to be extended to patients undergoing other types of abdominal surgery. Third, studies are warranted that focus on clinical reasoning and decision-making process in progressively mobilizing patients to maximize therapeutic response, particularly with respect to maximizing oxygen transport and minimizing negative sequelae of bed rest, while ensuring the patient is safe, and with a view toward long-term functional return.

## Conclusion

Compared with the initiation of mobilization the day after surgery, our findings support that judicious response-driven progressive mobilization initiated on the same-day as surgery improves oxygenation short-term in patients after open surgery for pancreatic cancer and is safe. Whether our findings predict reduced post-operative complications and hastened functional return warrants elucidation.

## Data Availability

The data sets used and/or analyzed during the current study are available from the corresponding author on reasonable request.
